# Towards Sustainable Composite Manufacturing with Recycled Carbon Fiber Reinforced Thermoplastic Composites

**DOI:** 10.3390/polym14061098

**Published:** 2022-03-09

**Authors:** Sarianna Palola, Pekka Laurikainen, Sonia García-Arrieta, Egoitz Goikuria Astorkia, Essi Sarlin

**Affiliations:** 1 Materials Science and Environmental Engineering, Faculty of Engineering and Natural Sciences, Tampere University, FI-33014 Tampere, Finland; pekka.laurikainen@tuni.fi (P.L.); essi.sarlin@tuni.fi (E.S.); 2 Tecnalia, Basque Research and Technology Alliance (BRTA), Mikeletegi Pasealekua, 2, E-20009 Donostia-San Sebastián, Spain; sonia.garcia@tecnalia.com; 3 Batz S.Coop, Automotive Division, Barrio Torrea 2, E-48140 Igorre, Spain; egoikuria@batz.es

**Keywords:** recycled carbon fiber, reactive pyrolysis, interfacial shear strength, thermoplastic, microbond

## Abstract

Currently, the vast majority of composite waste is either landfilled or incinerated, causing a massive burden on the environment and resulting in the loss of potentially valuable raw material. Here, conventional pyrolysis and reactive pyrolysis were used to reclaim carbon fibers from aeronautical scrap material, and to evaluate the feasibility of using reclaimed carbon fibers in structural components for the automotive sector. The need for fiber sizing was investigated as well as the behavior of the fiber material in macroscopic impact testing. The fibers were characterized with the single fiber tensile test, scanning electron microscopy, and the microbond test. Critical fiber length was estimated in both polypropylene and polyamide matrices. Tensile strength of the fiber material was better preserved with the reactive pyrolysis compared to the conventional pyrolysis, but in both cases the interfacial shear strength was retained or even improved. The impact testing revealed that the components made of these fibers fulfilled all required deformation limits set for the components with virgin fibers. These results indicate that recycled carbon fibers can be a viable option even in structural components, resulting in lower production costs and greener composites.

## 1. Introduction

Carbon fibers (CF) are lightweight reinforcing fibers with high specific strength and stiffness but also with high energy demand during the production stage. Due to their excellent properties, they are widely used in demanding applications, such as in the aeronautic sector [1,2], the automotive industry [3,4], and construction [5,6], with both thermoset and thermoplastic matrices. Widespread production and use of CF inevitably leads to high amounts of CF composite waste. From this perspective, thermoset composite waste is particularly troublesome. The majority of this waste is currently either incinerated or landfilled [7,8], even though landfilling has been stated to be the least preferable option for CF composite disposal by the European Waste Framework Directive [9]. From the circular economy point of view, it is not feasible to dispose of expensive and high-quality material, but rather to aim to re-use and recycle it. To solve this dilemma, several methods are used to reclaim CFs from composite waste, ranging from electromagnetic [10] to thermal [11,12,13,14] and thermochemical [15,16,17,18] methods. Depending on the method, either just the fiber material is reclaimed or also the matrix in a simultaneous process. Industrial manufacturers have also risen to the challenge, and for example, in the automotive sector, original equipment manufacturers (OEM) have a specific interest in using sustainable polymeric materials in thermoplastic injection molding compounds for structural components.

Thermoplastic polymers have excellent impact resistance, unlimited self-life, high strength and chemical resistance [4], which is why they are increasingly used in composite products. Polypropylene (PP) is one of the most often used commercial thermoplastic polymers in composite manufacturing, due to its low cost and simple processability, excellent mechanical and chemical properties, good thermal stability as well as low density. Polyamide 6 (PA6) is another widely used thermoplastic material with excellent abrasion resistance, heat resistance, and high mechanical strength [19].

The issue with CF and the thermoplastic matrix, from the composite point of view, is poor adhesion. The inert surface of CF and high viscosity of the thermoplastic matrix does not enable the formation of a strong interphase between the fiber and matrix, resulting in the loss of mechanical properties of the whole composite structure. To overcome this, various surface treatments [18] are used. The surface treatment is typically applied to the fiber surface as a part of the initial fiber manufacturing process. However, when considering recycled fiber material, the original sizing is removed simultaneously with the matrix material during the recycling process. Resizing of the rCFs cannot be done in the same fashion as during the fiber manufacturing process, since the rCF material is often chopped or shredded during the recycling process. A different approach must be adopted when considering the rCF material. For example, Fernandez et al. [20] used a hot-press to resize pyrolytically reclaimed rCFs with a commercial maleic anhydride grafted polypropylene (MAH-PP) resin. However, they noticed that even though this approach resulted in improved overall composite properties, it was mainly due to the low-crystallinity MAH-PP reacting with the PP matrix. This leads to the question if resizing of the rCFs is necessary in the first place. Cai et al. [21] addressed this by studying the effect of recycling parameters on interfacial shear strength (IFSS) of rCF in the PP matrix. They used super-heated steam as the recycling method and discovered that with optimum process parameters, the surface chemical state of the rCFs is compatible with PP. This way, the need for resizing could be eliminated altogether. Overall, relatively few studies have been done with long (length in mm scale) rCF in a thermoplastic matrix, and even fewer, where the microscale interfacial properties and macroscale composite properties are investigated side-by-side.

In this study, the effects of recycling method on the tensile and adhesive properties of rCFs are investigated. The aim is to determine how well fiber properties are preserved during the recycling process. Another goal is to study how the interfacial properties of rCFs compare to virgin CF in thermoplastic matrices in the microscale and how they translate to macroscale properties in structural injection molded components. The fibers were reclaimed with two different thermal methods: conventional pyrolysis and modern reactive pyrolysis. The resulting rCFs were resized to see if resizing was necessary. Scanning electron microscopy (SEM) was used to study the changes in the surface structure and topography of the fibers. Single fiber tensile testing was used to verify the tensile integrity of the fibers together with changes in the fiber diameter. Critical length for the fibers was calculated to gain an understanding of how the fibers will behave in a composite form. To study the adhesive properties of the fibers in PP and PA6 matrices, a high throughput microbond method was used. To demonstrate the applicability of the recycled carbon fibers in industrial environment, two use cases were selected from automotive industry applications. Macroscopic demonstrator pieces were manufactured by injection molding. Impact testing and SEM investigation were used to study the properties of these composite demonstrators and to see how the fiber–matrix interphase is formed. The impact and microbond test results were compared to investigate how well the microscale results are translated to macroscopic behavior.

## 2. Materials and Methods

### 2.1. Carbon Fibers

Recycled carbon fibers used in this study were polyacrylonitrile-based carbon fibers reclaimed from cutting scraps and outdated pieces of uncured CF/epoxy prepreg from the aeronautical sector. This waste material was chosen for the project as it was readily available, but any end-of-life composite product or piece could be used to reach the same outcome. Reclaiming/recycling was done with two pyrolytic methods: conventional pyrolysis, and reactive pyrolysis. Conventional pyrolysis was performed in an inert nitrogen atmosphere in an oven with a total cycle time of 6 h with heating ramps of 200 °C/h until 450 °C. These CFs recycled with the conventional pyrolysis are denoted as rCR_p_. The reactive pyrolysis, on the other hand, was done in a controlled CO_2_ environment as a thermochemical depolymerization process [18]. This way, both the inorganic part (carbon fibers) and the organic part (resin) could be recovered simultaneously, the latter in the form of an organic liquid fraction. Prior to the reactive pyrolysis process the waste material was shredded mechanically with blade mills to 10–15 mm pieces. CFs recycled with this reactive method are denoted as rCF_s_. As a reference, a virgin carbon fiber (Toho Tenax America Inc., Rockwood, TN, USA), denoted as CF, was used, which had similar original properties of tensile strength 4500 MPa, tensile modulus 240 GPa, and nominal linear density 800 tex. However, it is noteworthy that these properties reported by the manufacturer are for a fiber bundle and not for a single fiber filament. The CFs were washed with two consecutive ultrasonic acetone baths (2 × 5 min), to remove surface sizing from the fibers.

### 2.2. Matrix Materials

For the micro and macroscale testing, both polypropylene (PP) and polyamide (PA6) matrices were used. The PP was a high melt flow heterophasic copolymer (tensile modulus 1300 MPa, tensile stress at yield 25 MPa, density 905 kg/m^3^), and the PA6 a low-viscosity polyamide (tensile modulus 2300 MPa, tensile stress at yield 80 MPa, density 1130 kg/m^3^). Both polymers were received in granulate form. Due to the confidentiality of the related project, more detailed information about the matrices cannot be provided.

### 2.3. Fiber Surface Modification

A non-ionic malleated polypropylene (MAPP) dispersion (Hydrosize^®^ PP2-01, Michelman Inc., Cincinnati, OH, USA) was used with the PP matrix as the film former (FF), whereas a non-ionic polyurethane (PU) dispersion (Hydrosize^®^ U2022, Michelman Inc., Cincinnati, OH, USA) was used as film former with the PA6 matrix. The rCF resizing was done by preparing 1 wt% and 5 wt% solids content solutions of each film former with deionized water. Each of the solutions was heated up to 30 °C and kept at this temperature for 1 h with continuous mixing. The rCFs were immersed into each of the solutions and then rinsed with deionized water to remove excess sizing. Finally, the fibers were dried in a convection oven at 80 °C for 18 h.

For the macroscopic demonstrators, a larger batch of the resized rCF_s_ was prepared using the 5 wt% solutions of the PP and PU dispersions for both PP and PA matrices, respectively. The stronger concentration was chosen as it performed the best with the pristine CF (see Section 3) and enabled easier handling of fiber material during compounding. The fiber material was dipped in the sizing solution, and then excess solution was squeezed out manually, after which they were placed into a convection oven kept at 80 °C until fully dry. Rinsing was omitted to ensure suitable specific density of the resized fibers for successful feeding and compounding.

### 2.4. Interfacial Shear Strength

IFSS was measured with the microbond test method [22], in which single fiber microcomposite samples were prepared and tested. The samples were prepared by depositing droplets of the matrix material onto single fiber filaments and allowing them to harden. The droplets were then individually loaded with microvise blades (loading rate 0.008 mm/s) until the droplet detached from the fiber. To calculate the apparent IFSS (ΔIFSS) for each droplet, the load required to detach the droplet (F_max_) was compared with the area of the fiber surface embedded by the said droplet (A_emb_), as described by Equation (1). A linear fit was then applied to all of the data points acquired this way from each fiber to calculate the IFSS of the said fiber.

(1) $$\frac{F_{\max}}{A_{\mathrm{emb}}}=\Delta \mathrm{IFSS}$$

PA6 and PP droplets were dispensed onto the fibers with a FIBROdrop (Fibrobotics Oy, Tampere, Finland) setup. A computer-controlled heating element was used to achieve optimum melt flow during droplet deposition. To prevent oxidation and thermal degradation of the polymer melt during the droplet deposition, the FIBROdrop device was placed into an air-tight cabinet filled with nitrogen (N_2_) gas. In addition, a new batch of polymer melt was prepared for each fiber.

The microbond measurements, in this study, were done with a high-throughput FIBRObond (Fibrobotics Oy, Tampere, Finland) [23] device. The load cell used was varied depending on the matrix: a 1 N load cell with PA6, and 0.1 N load cell with PP. For testing, the fiber samples were attached to stainless steel sample holders. Testing was done in ambient laboratory conditions. Five fibers per sample type with 20–40 droplets per fiber were tested, resulting in approximately 100–200 data points for each sample type.

### 2.5. Single Fiber Tensile Testing

Single fiber tensile testing was done with a FIBROtensile (Fibrobotics Oy, Tampere, Finland) system under ambient laboratory conditions. Prior to testing, fiber diameter was recorded with the measurement system’s optical microscope for each fiber filament separately. The testing was performed with a 1 N load cell and gauge length of 23.6 mm. Apart from the standard ISO 5079:1995, a slower loading rate of 0.008 mm/s was chosen due to the delicate nature of the rCF fibers; 30–50 fibers per sample set were tested.

### 2.6. Scanning Electron Microscopy

A Zeiss ULTRAplus (Zeiss, Oberkochen, Germany) field emission gun SEM was used for detailed imaging of the fibers and of the failed fiber–matrix interphases after micro- and macroscale testing. To minimize charging and to improve image quality, the microscale test samples were attached to aluminum sample holders with carbon tape and coated with a ~3 nm layer of palladium and platinum (ratio: Pt/Pd 80/20 wt%) mixture. Polished cross-sectional samples were prepared from the macroscale use case components in addition to the fracture surfaces. The cross-sectional samples were taken parallel to the fiber direction to allow evaluation of the fiber length. However, as the exact orientation of the fibers is challenging to define from an injection molded structure, some deviation from the actual fiber length were expected towards shorter lengths. The fiber length was calculated from 100 random fibers based on the SEM image analysis done with the open-source software ImageJ (https://imagej.nih.gov/ij/, accessed on 28 December 2021).

### 2.7. Use-Case Study

Two use-cases were explored: a pedal bracket, and a front-end carrier. The pedal bracket is a component in which gas and brake pedals are assembled in a vehicle. It must meet high structural requirements to ensure the safety of the vehicle, especially in emergency situations. Currently, there are lightweight glass fiber reinforced thermoplastic solutions for pedal brackets on the market, while traditionally a metallic solution is used. Therefore, a virgin glass fiber reinforced PA6 compound (fiber fraction 40 wt%) was used as the reference material for the PA6 rCF_s_ compound (fiber fraction 20 wt% ± 2 wt%) used in this study.

The front-end carrier is a frontal structure of the vehicle for the assembly of other components, such as highlights, radiator, and latches. It has to support the weight of the attached components and contribute to the torsion performance of the car. The modern lightweight solutions are made of high fiber fraction (up to 40–50 wt%) reinforced thermoplastics, and in this study, a PP compound with long virgin glass fibers (fiber fraction 40%) was used as a reference material for the PP rCF_s_ compound (fiber fraction 20 wt% ± 2 wt%). In both use cases, additional motivation for the use of recycled feedstock material is the reduced amount of used reinforcement and the reduced weight of the final component with the glass to carbon transition.

The resized rCFs were fed to a hopper and compounded using a Coperion Werner & Pfleiderer ZSK 26 P 10.6 twin screw extruder with a Brabender feeder for the matrix material and a double screw side feeder for the fibers. As a part of the compounding process, the materials were pelletized. The final fiber fraction of the compounds was verified with a thermogravimetric analysis (TGA, TA Instrument Universal V4, New Castle, DE, USA).

Both of the use-case components were prepared to their actual geometry with industrial processes: classic thermoplastic injection process for the pedal bracket, and injection molding compound (IMC) process for the front-end carrier. The injection process was similar for the recycled and the reference materials, but some parameters, such as process temperatures or injection velocity, had to be adapted to the rheological characteristics of the recycled material. The components were mechanically tested based on OEM standards and compared with the respective components made with the reference materials.

The industrial validation tests for the use case components were based on the typical loading cases of the components in question. For the pedal bracket, the component was first subjected to conditioning cycles with hot and humid phases (80 °C and 80% RH) but also cold ones (−40 °C and 30% HR). Each complete cycle took 12 h and needed to be repeated 8 times (equal to 4 days). The pedal bracket was then subjected to loads applied to the brake and gas pedals assembled into the bracket at different temperatures (−35 and 80 °C). Two load levels were used for both pedals (see Table 1). For the lower loads, there are limits for accepted elastic and plastic deformations of the pedals defined by the vehicle manufacturer, whereas for the higher loads, the validation was based on possible permanent failure/damage of the component. The validation limits were the same for both test temperatures. For the front-end carrier, the validation test focused on static traction and compression loading of the bonnet latch with specific limits for the strength and stiffness. Only one load level was used in the tests. As the exact process parameters, component geometry, or test geometry details of the components are not public, the results of mechanical testing were compared only qualitatively to understand if there is industrial potential for recycled carbon fibers.

## 3. Results and Discussion

### 3.1. Fiber Characterization

**Figure 1:**
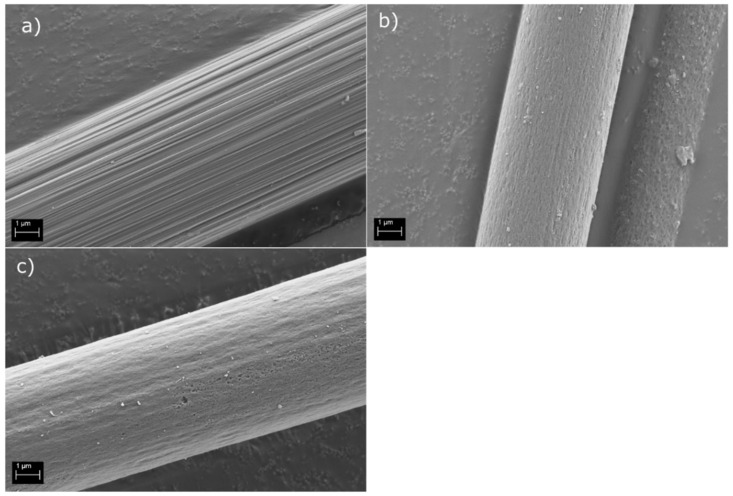
SEM images of (**a**) washed CF, (**b**) rCFs, and (**c**) rCFp.

The characteristic grooves of CF were clearly visible on the pristine CF surface (Figure 1a), and the surface appeared overall clean and free of sizing residue, indicating a successful washing procedure. As expected, the recycling processes altered the topography of the rCF_p_ and rCF_s_ surfaces (Figure 1b,c). For example, the parallel grooves running along the fiber surface were more subdued in the rCF_s_ and rCF_p_. In addition, there was a clear difference between the two recycled fiber surfaces. The rCF_p_ surface appeared smoothed out and clearly degraded when compared to the rCF_s_ surface, which retained some of the grooved surface characteristic typical of CFs. There was also some evidence of pitting on the rCF_p_ surface. This was anticipated, as the conventional pyrolysis process is known to be a harsh treatment for the fibers. The rCF_p_ surface also had some matrix residue and debris left over from the pyrolysis process. The rCF_s_ surface appeared cleaner, indicating successful matrix removal.

**Figure 2:**
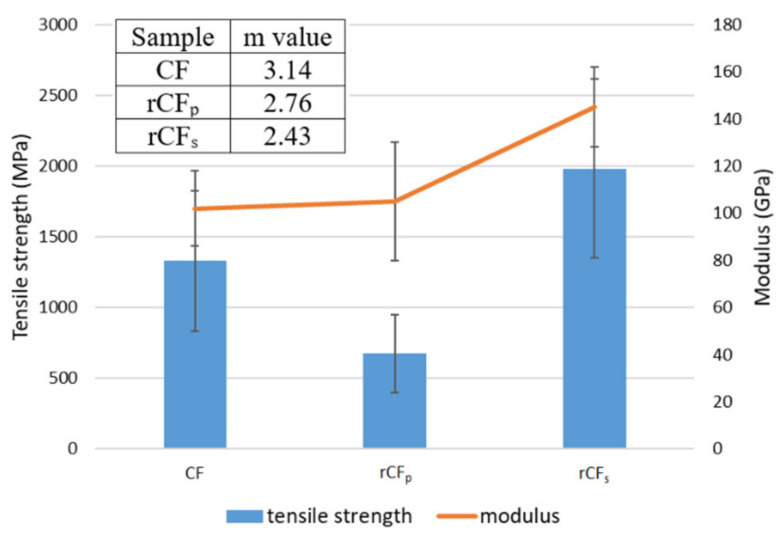
Tensile strength and modulus of CF, rCF_p_, and rCF_s_ and the corresponding shape parameter m.

Single fiber tensile test results are presented in Figure 2, including the Weibull shape parameter m. The results show that the tensile strength of the pyrolytically reclaimed rCF_p_ (670 MPa) was significantly lower than that of the rCF_s_ (1980 MPa). This confirms the notion of fiber damage due to the harsh recycling process, as was suggested by the SEM image in Figure 1c. On the other hand, the tensile strength of the rCF_s_ fibers was similar to that of the pristine CFs (1330 MPa), indicating that the method was not as detrimental to the fiber properties. The rCF_s_ achieved higher tensile values than the CF because the original reclaimed CF was not the same as the one used as reference in this study. The fiber properties of the two were, however, a very close match, enabling the qualitative comparison of results.

Interestingly, the moduli of all three sample types were quite similar, with rCF_s_ showing only slightly (~40 GPa) higher values. Additionally, fiber diameter (6.4 µm) remained the same for both the rCF_p_ and rCF_s_, despite the large difference in the tensile strength values. Pitting on the fiber surface (Figure 1c) was most likely a major contributing factor to the poor tensile properties of the rCF_p_. The crevices and holes on the fiber surface may have acted as initiation sites for fractures during tensile loading and thus resulted in catastrophic failure of the fiber at lower load values. This is further supported by the Weibull shape parameter m [24,25] in Figure 2. The higher m value of 3.14 for the CF represented a fairly symmetric damage probability distribution, as expected of a virgin fiber, whereas for the recycled fibers, the lower shape parameter values implied varying degrees of non-uniformity in the structure. This likely arose from the damage of the fiber structure due to the recycling process, such as the aforementioned pitting.

### 3.2. Interfacial Shear Strength

**Figure 3:**
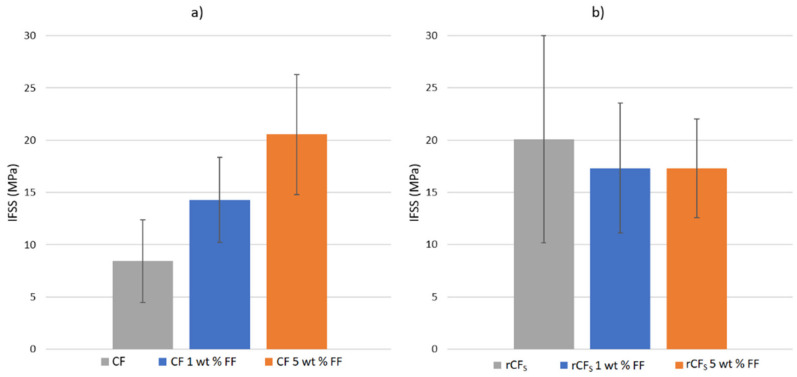
IFSS test results for (**a**) pristine CF and PP and (**b**) recycled rCF_s_ and PP.

The IFSS of CF in PP was measured to be 8.4 MPa, which corresponds well with values reported in the literature [21]. When applying a 1 wt% surface sizing solution to the fiber surface, the IFSS increased to 14.3 MPa, as seen in Figure 3a. With a stronger 5 wt% sizing solution, the IFSS increased further to 20.5 MPa. These results show that by applying a sizing layer to the virgin fiber surface, IFSS was indeed improved, and a stronger interphase was formed between the fiber surface and matrix.

The increase in IFSS was because of the co-polymer character of MAPP sizing. The hydrophilic anhydride groups of the sizing reacted with the hydroxyl groups on the CF surface, while the hydrophobic PP section was compatible with the PP matrix molecules [26]. This resulted in a stronger interfacial interaction and thus stronger adhesion with the matrix. In addition, the sizing solution helped to wet the fiber surface with the viscous polymer melt during droplet deposition, resulting in enhanced interfacial interaction. It is clear that the resizing concept is a viable method to enhance adhesion at the fiber–matrix interphase in cases where the original manufacturing related sizing has been removed.

**Figure 4:**
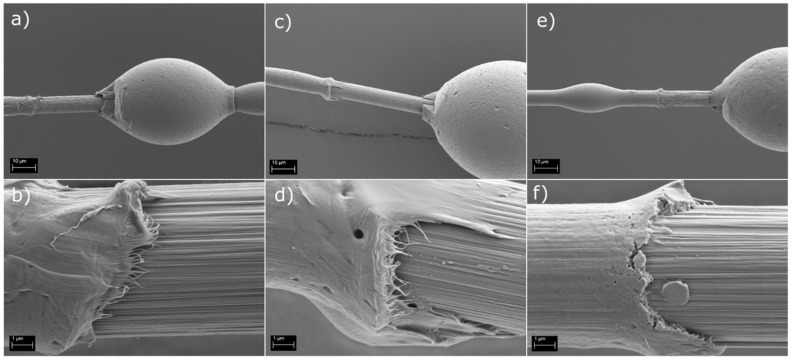
Fracture surfaces of (**a**,**b**) CF, (**c**,**d**) CF + 1 wt% FF solution, and (**e**,**f**) CF + 5 wt% FF solution, in the PP matrix.

**Figure 5:**
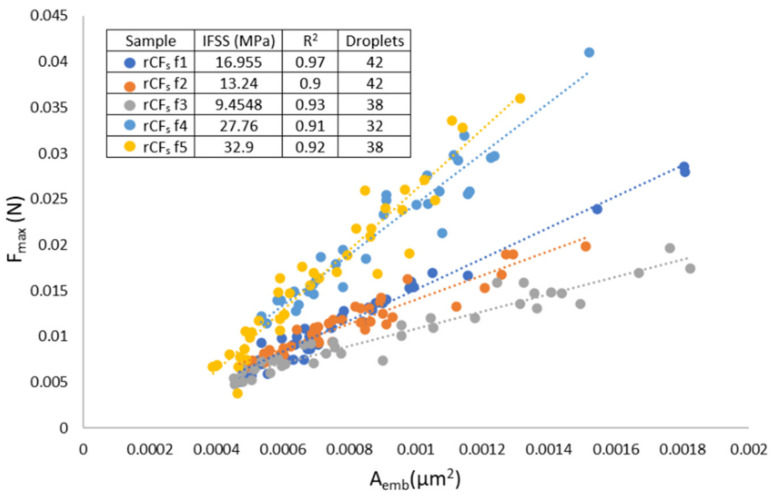
Individual data points for the rCF_s_ fibers as a function of embedded area.

The SEM images in Figure 4 support this notion. It can be seen that the PP matrix was more strongly bonded to the CF fiber when 1 wt% (Figure 4c,d) and 5 wt% (Figure 4e,f) sizing solutions were applied, as is suggested by the IFSS results. The un-sized fiber surface (Figure 4a,b) had a clear gap between the matrix and fiber surface at the debonding site. In addition, the detached fiber surface was clean, with no matrix residue left from the detached PP droplet. However, the amount of adherent matrix residue increased with the application of the surface sizing and even more so as the sizing solution concentration increased. In addition, the gap between fiber and matrix disappeared and was replaced with a strong interphase, even after droplet detachment, as seen from Figure 4e,f.

However, when considering the IFSS results of the recycled fibers, the application of the surface sizing did not appear to have the same effect, as seen in Figure 3b. On the other hand, the un-sized rCF_s_ samples already had the same “increased” IFSS value of 20.1 MPa, similar to the CF samples sized with 5 wt% sizing solution. It is also noteworthy that the IFSS of the rCF_s_ was higher than the previously [21] reported values for un-sized rCF and PP. Applying 1 wt% or 5 wt% sizing solutions to the rCF_S_ fibers did not increase the IFSS any further. The IFSS of rCF_s_ resized with 1 wt% and 5 wt% solids content FF solutions was 17.3 MPa for both. The exact value appeared slightly lower than that of the un-sized rCF_s_ but was well within the deviation range, as seen from Figure 3b. The increased surface topography of rCF_S_ was most likely the main contributor to the enhanced IFSS, as noted in other similar studies [19,27,28,29]. The surface of both of the rCF types (conventional and reactive pyrolysis) was more textured and rougher in the loading direction than of the pristine CFs (Figure 1). This increased the frictional forces and mechanical adhesion at the interphase and thus also the IFSS. In addition, the fiber surface was most likely oxidized during the reactive pyrolysis, which is known to contribute favorably to the interfacial adhesion [30]. The application of resizing, on one hand, smoothened out the fiber surface but also simultaneously enhanced the adhesion by increasing hydrogen bonding at the surface. Hence, no dramatic change in the IFSS was noted after the resizing process. The IFSS of rCF_p_ was measured to be similar to the rCF_s_ at 21.3 MPa (stdev 4.9 MPa) owing to the pronounced surface topography. However, no resizing trials were done with it because of its poor tensile properties.

In Figure 3b, the larger deviation of the un-sized rCF_s_ IFSS results was because one of the measured fibers (rCF_s_ f3, see Figure 5) had lower IFSS than the bulk of the results. As seen from Figure 5, all of the measured fibers had excellent linear fits, with R^2^ values over 0.9. This means that the measurement and the results were highly accurate and represented the data very well. In fibrous material, systematic deviation occurs naturally along the fiber length [23]. This natural variation in the fiber surface properties was visible in the CF results as well, but it was more pronounced in the rCF_s_ results due to changes in the fiber surface structure induced by the recycling process. This phenomenon will naturally further contribute to the larger overall deviation.

The IFSS results for CF and rCF_s_ in PA6 are presented in Table 2. From the results it can be seen that the values for both fiber types were similar. The values for both were also notably higher than what have been reported elsewhere [22,31] with untreated CF and PA6. This is significant, as it indicates that an excellent level of interfacial adhesion was achieved with the rCF_s_ in the thermoplastic matrix. In addition, as the IFSS of CF was already very high, testing with resized CF was omitted.

When PU FF was applied to the rCF_s_ samples, it appeared that the IFSS decreased. However, this was not the case, but rather an inconsistency caused by fibers failing during the microbond testing. The application of the PU FF increased the IFSS of the rCF_s_ to a level that was higher than the cohesive strength of the fiber. This caused the apparent decrease in the IFSS of rCF_s_ 1 wt% FF samples, as the only successful measurements could be achieved from fibers with a poor interphase. From Table 2 it can be seen that the number of fiber samples needed to achieve five successfully measured fibers increased as PU sizing was applied. The phenomenon was even more pronounced with the rCF_s_ 5 wt% FF samples, as the fibers under testing would break even at the very smallest PA6 droplets (~25 µm embedded length), thus preventing the accumulation of enough data points of different size scales for the linear fit to be applied. On the other hand, qualitative analysis of the results showed that the IFSS in PA6 increased when PU FF was applied to the fiber surface.

Reinforcing efficiency of fibers in a fiber reinforced plastic (FRP) compound is not only dependent on the interfacial adhesion between the fibers and matrix but also on the length of the reinforcing fibers, called critical fiber length. If the critical fiber length is exceeded, fiber breakage becomes a notable damage propagation process in addition to matrix and interfacial failure, limiting further improvement of the composite properties (i.e., load bearing capability). Thus, by calculating the critical fiber length in any given FRP system, it is possible to evaluate the reinforcing ability of the fibers in the said system. This is important especially in short fiber composites, such as those made of rCFs. The critical fiber length ($L_{c}$) for the CF + PP/PA6 and rCF +PP/PA6 was evaluated with Equations (2) and (3) [21]:

(2) $$L_{c}=\frac{\sigma_{f}\left(L_{c}\right)D_{f}}{2\tau }$$

(3) $$\sigma_{f}\left(L_{c}\right)=\sigma_{0}{\left(\frac{L_{0}}{L_{c}}\right)}^{\frac{1}{m}}\Gamma \left(1+\frac{1}{m}\right)$$

where $\sigma_{f}\left(L_{c}\right)$ is the fiber strength at critical length, (Γ) the gamma function, (m) the Weibull modulus, ($\sigma_{0}$) the characteristic stress, and ($L_{0}$) the reference length. Critical length values obtained with the formula for each of the fiber types in both PP and PA6 matrices are presented in Table 3.

From these results, it can be seen that the reinforcing potential of the rCFs was quite good as the critical length was smaller than with the CF in the PP matrix and very close to it in the PA6 matrix. This is an important notion, as the fiber length is often dramatically reduced during post-processing procedures, such as compounding, that are used typically with rCF material and the thermoplastic matrix. The above reported values are similar to values reported elsewhere [21], supporting the notion of rCF material as a viable option for composite manufacturing.

### 3.3. Macroscopic Testing of Use-Case Applications

**Figure 6:**
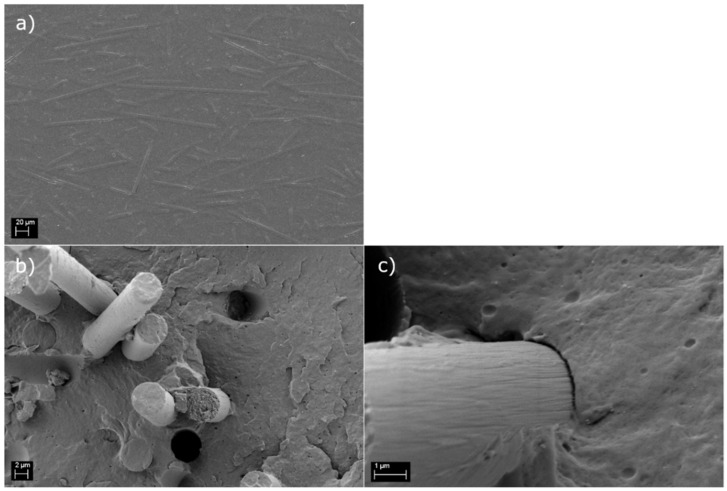
Polished cross-section (**a**) and fracture surface (**b**,**c**) of the PA6 rCFs compound.

The pedal brackets made of PA6 and the rCF_s_ and reference materials fulfilled all deformation limit requirements without suffering any damage, demonstrating the technical viability of recycled carbon fibers to replace virgin glass fibers in the automotive industry. The cross-sectional SEM studies revealed a high-quality composite material (Figure 6a) and an average fiber length (±standard deviation) of 90 ± 52 μm, which is well below the obtained critical length value. This is important, as is confirms that the full reinforcing potential of the fibers is utilized. The fracture surface investigation (Figure 6b,c) ensured that the interphase between the sized recycled carbon fibers and the PA6 matrix was good, as expected based on the mechanical validation tests. Even though some pull-outs were visible, the fibers clearly had matrix residue sticking to their surface, indicating a combined failure of the interphase and matrix.

**Figure 7:**
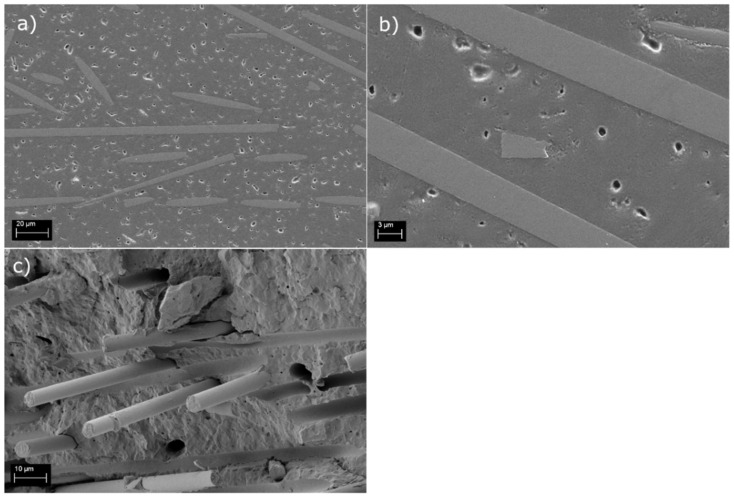
Polished cross-section (**a**,**b**) and fracture surface (**c**) of the PP rCFs compound.

The front-end carrier made of the rCF_s_ and PP fulfilled the set requirements and exceeded the properties of the reference material in all tests, excluding the strength values under traction loading. For that specific value, the results were 34.6% lower than for the reference material, although they met the set test limit. The cross-sectional SEM samples exhibited a porous structure (Figure 7a,b), explaining the lower strength values obtained in mechanical tests. Clearly, the injection molding process of the recycled material was not optimal, despite the process parameter adjustment. The average fiber length (±standard deviation) was measured to be 89 ± 67 μm. In addition, in this case, the fiber length in the actual composite was smaller than the estimated critical fiber length. This indicates that the full reinforcing potential of the rCFs was utilized in this use-case component as well. Similar to the PA6 rCF_s_ compound, the fracture surface study (Figure 7c) verified a good quality fiber/matrix interphase with limited amount of pull outs.

These results combined with the IFSS results demonstrate that the rCFs contributed profoundly to the overall performance of the composite. This is a significant find, as in another study [20], a question was proposed regarding the reinforcing efficiency of rCF. They concluded that the major factor in improving the impact resistance of rCF + PP compounds would be the addition of surface sizing, which toughens the matrix rather than improves the fiber–matrix interphase. However, our IFSS results, and critical length calculations combined with the macroscopic impact testing strongly highlighted the importance of a strong fiber–matrix interphase. This way, the full reinforcing potential of rCF material can be utilized in structural composite components.

## 4. Conclusions

In this study, two different recycling methods were used to reclaim rCFs. Strong and clean recycled carbon fibers were acquired with the reactive pyrolysis process, whereas conventional pyrolysis resulted in fibers with poor tensile strength. However, the IFSS of both of these fibers was highly comparable to those of virgin carbon fibers in PP and PA6 matrices. Resizing of the fibers was also investigated, and it can be considered not to be necessary from the adhesion point of view due to the favorable surface structure of the rCFs. However, for post-processing, such as compounding and injection molding, it is vital to ease the handling of the fiber material and to ensure the production of high-quality composite products. Impact testing of macroscopic use-case demonstrators revealed that the recycled carbon fibers are indeed a valid option for virgin carbon fibers, in terms of durability and toughness.

Thus, our recommendation is that in the future, research in this field should be directed towards developing recycling methods to be more economical, rather than to fiber property optimization, since fiber integrity and adhesion properties can already be achieved, especially with short fiber compounds. With a suitable recycling method, such as the reactive pyrolysis process used in this study, the recycled carbon fibers do not portray the same adhesion issues with PP or PA6 matrices as virgin carbon fibers do.

## Figures and Tables

**Table 1:** Validation test cases for the macroscale use case components.

Use Case	Loaded Component	Load (kN)	Deformation Limits	
			Elastic Deformation (mm)	Plastic Deformation (mm)
Pedal bracket	Brake pedal	2.3	<17.0	<5.0
		3.0	No failure	No failure
	Gas pedal	0.2	<6.0	<2.0
		1.0	No failure	No failure
			**Stiffness (N/mm)**	**Strength**
Front-end carrier	Bonnet latch traction	2.3	>420	No failure
	Bonnet latch compression	1.5	>350	No failure

**Table 2:** IFSS results and standard deviation (stdev) of CF and rCFs in PA6. The last column presents the number of successfully tested fiber samples versus fibers that failed during testing.

Sample	IFSS (MPa)	Stdev (MPa)	Successful/Failed Fiber Samples
CF	62.5	3.7	5/0
rCF_s_	66.5	6.0	5/1
rCF_s_ 1 wt% FF	54.7	5.5	5/3
rCF_s_ 5 wt% FF	N/A	N/A	0/5

**Table 3:** Comparison of the critical length of un-sized CF, rCF_s_, and rCF_p_ in both PP and PA6 matrices.

Sample	Matrix	Critical Length (mm)
CF	PP	2.00
	PA6	0.51
rCF_s_	PP	1.67
	PA6	0.78
rCF_p_	PP	0.46

## Data Availability

The data presented in this study are available upon request from the corresponding author.
